# HIV test-and-treat policy improves clinical outcomes in Zambian adults from Southern Province: a multicenter retrospective cohort study

**DOI:** 10.3389/fpubh.2023.1244125

**Published:** 2023-10-11

**Authors:** Benson M. Hamooya, Simon Mutembo, Brian Muyunda, Keith Mweebo, Nzali Kancheya, Lyapa Sikazwe, Morgan Sakala, Johanzi Mvula, Salazeh Kunda, Shem Kabesha, Chilala Cheelo, Isaac Fwemba, Clive Banda, Sepiso K. Masenga

**Affiliations:** ^1^School of Medicine and Health Sciences, Mulungushi University, Livingstone, Zambia; ^2^International Vaccine Access Center, Department of International Health, Bloomberg School of Public Health, Johns Hopkins University, Baltimore, MD, United States; ^3^Centers for Disease Control and Prevention, Lusaka, Zambia; ^4^Provincial Medical Office, Ministry of Health, Choma, Zambia; ^5^School of Public Health, University of Zambia, Lusaka, Zambia

**Keywords:** retention, HIV, testing, antiretroviral therapy, Zambia, clinical outcomes, test-and-treat, Southern Province

## Abstract

**Background:**

Globally, most countries have implemented a test-and-treat policy to reduce morbidity and mortality associated with HIV infection. However, the impact of this strategy has not been critically appraised in many settings, including Zambia. We evaluated the retention and clinical outcomes of adults enrolled in antiretroviral therapy (ART) and assessed the impact of the test-and-treat policy.

**Methods:**

We conducted a retrospective cohort study among 6,640 individuals who initiated ART between January 1, 2014 and July 31, 2016 [before test-and-treat cohort (BTT), *n* = 2,991] and between August 1, 2016 and October 1, 2020 [after test-and-treat cohort (ATT), *n* = 3,649] in 12 districts of the Southern province. To assess factors associated with retention, we used logistic regression (xtlogit model).

**Results:**

The median age [interquartile range (IQR)] was 34.8 years (28.0, 42.1), and 60.2% (*n* = 3,995) were women. The overall retention was 83.4% [95% confidence interval (CI) 82.6, 84.4], and it was significantly higher among the ATT cohort, 90.6 vs. 74.8%, *p* < 0.001. The reasons for attrition were higher in the BTT compared to the ATT cohorts: stopped treatment (0.3 vs. 0.1%), transferred out (9.3 vs. 3.2%), lost to follow-up (13.5 vs. 5.9%), and death (1.4 vs. 0.2%). Retention in care was significantly associated with the ATT cohort, increasing age and baseline body mass index (BMI), rural residence, and WHO stage 2, while non-retention was associated with never being married, divorced, and being in WHO stage 3.

**Conclusion:**

The retention rate and attrition factors improved in the ATT compared to the BTT cohorts. Drivers of retention were test-and-treat policy, older age, high BMI, rural residence, marital status, and WHO stage 1. Therefore, there is need for interventions targeting young people, urban residents, non-married people, and those in the symptomatic WHO stages and with low BMI. Our findings highlight improved ART retention after the implementation of the test-and-treat policy.

## 1. Introduction

Increasing early access to antiretroviral therapy (ART) in resource-limited settings has improved the lives of people with HIV (PWH) in Africa (1, 2). Several strategies have been implemented to reduce morbidity and mortality among PWH. Among these strategies is the test-and-treat approach, where ART is initiated soon after diagnosis of HIV infection following the World Health Organization (WHO) recommendations of 2015 (3). Implementation of this universal test-and-treat policy was projected to prevent at least 21 million deaths and 28 million new infections by 2030 globally (4). In 2016, the Zambia Ministry of Health started implementing the universal HIV test-and-treat policy for all PWH, regardless of their CD4 count or WHO HIV clinical stage at the time of diagnosis. However, some scientists and program managers were concerned about the approaches to use to initiate ART for the newly diagnosed and supposedly healthy, to handle issues of HIV stigma, manpower, and infrastructure constraints, and to provide psychosocial counseling and clinical follow-up for large numbers of PWH (5–7).

However, the beneficial effects of early ART initiation are overwhelming in the prevention and treatment of HIV infection. Universal testing and treatment (UTT) has been linked to a reduction in HIV-associated morbidity and mortality (8–11). In the presence of optimal adherence, early initiation of ART leads to rapid attainment and sustained HIV viral suppression (8, 11–14). This is critical to the reduction of HIV transmission at the individual (15) and community (16, 17) levels and to achieving the UNAIDS 2030 target of 95-95-95 (18). The effect of an early ART start has also been observed in sub-Saharan Africa (SSA). Retention in care increased in Malawi (19) and Zambia (20), and reduced mortality was observed in the studies conducted in Uganda (21) and South Africa (22). Despite the well-documented benefit of UTT, other studies in Africa have shown an increase in loss to follow-up (LTFU) (23–25) and a decrease in ART retention in the first year (21, 26, 27). Some scholars have attributed the problems with retention and LTFU to a lack of realization of the beneficial effects of ART when individuals are initiated while enjoying a healthy state (23). Additionally, the lack of special support for individuals during UTT has been implicated in low retention rates (28).

For countries in SSA to achieve the sustainable development goal (SDG-3.3) of ending the AIDS epidemic by 2030 (29), retention in care and sustained viral suppression are critical. According to WHO, SSA has a substantial challenge with long-term retention in care (30). Persons who disengage from care and stop taking medication have a high probability of transmitting HIV infection, attaining AIDS status, and dying (30). The drivers of retention in care include female sex, older age, disclosure of HIV status, optimal ART adherence, viral suppression, a high CD4 count, and delayed ART initiation after HIV diagnosis (19, 26, 31, 32). Being divorced and in a relationship, acquiring HIV infection perinatally, and voluntary testing and counseling are some of the drivers of retention (33–35) in our setting. Therefore, there is an urgent need for locally tailored empirical evidence on the drivers of retention to attain HIV epidemic control in SSA.

In view of the existing literature, several studies in different settings have demonstrated the beneficial effect of the test-and-treat strategy (13, 14, 36) but with inconsistent findings on retention in care and other clinical outcomes such as death and LTFU. However, the impact of this strategy has not been adequately evaluated in a programmatic setting like Zambia, where the prevalence of HIV is high. We, therefore, conducted a multicenter retrospective cohort study to determine the effect of HIV test-and-treat on clinical outcomes of adults initiated on ART before and after implementation of the test-and-treat policy, with the hypothesis that the test-and-treat policy improved the HIV retention rate.

## 2. Methods

### 2.1. Study design

We conducted a retrospective cohort study of PWH enrolled in HIV care in 12 President’s Emergency Plan for AIDS Relief (PEPFAR)-supported districts in one province of Zambia.

### 2.2. Study population

Clinical data from patient records were extracted for PWH aged 15 years and older who were initiated on ART between January 1, 2014 and October 1, 2020, regardless of their clinical outcome at the time of data abstraction. PWH initiated on ART before the launch of the test-and-treat policy (January 1, 2014 to July 31, 2016) formed the pre-test-and-treat cohort, and those initiated in the period following the implementation of the test-and-treat policy (August 1, 2016 to October 1, 2020) formed the post-test-and-treat cohort.

All PWHs that enrolled in care but were not initiated on ART by the time of data abstraction were excluded from the analysis. However, the details of those who were not on ART were provided to the ART health providers for further follow-up and counseling with the aim of commencing ART. PWH with either a missing date of birth or age or a missing ART initiation date were excluded from the study.

### 2.3. Data collection and study procedures

Clinical data for PWH were abstracted either from HIV paper-based or electronic medical records known as SmartCare in 45 health facilities located in 12 districts of Southern Province onto the Electronic Data Capture (REDCap) software. Patient outcomes were recorded around the follow-up period of 3, 6, 12, and 24 months ±30 days and the last date of contact with the health care provider. In addition to clinical data (ART regimen, WHO stage, BMI, duration on ART, retention, stopped treatment, death, transferred out, and LTFU), laboratory (CD4 count and HIV viral load), and pharmacy-related data, including medicine refill dates, were collected from laboratory and pharmacy registers, respectively, in facilities where SmartCare was not fully functional. Where possible, the data were triangulated from both SmartCare and paper registers.

A line list of PWH was created from the ART clinic enrollment register per national guidelines. If the site had fewer than 100 patients on ART, data were abstracted from all the patient SmartCare medical records. For sites with more than 100 patient records, a systematic sampling scheme was used. At each site, we determined a sampling interval (X) by dividing the total number of client records at the site by the sample size and taking the whole number only (dropping the decimals); this process was done for the allocated sample sizes in the before and after test-and-treat strategies. The first record for review was selected (N) by randomly selecting a number between 1 and X (sampling interval). Data abstractors selected the Nth record and proceeded with selecting record N + X as the first consecutive record for review, which continued with adding X until the quota for that site had been reached. All the selected records were entered into the REDCap application and screened for inclusion and exclusion criteria. If any patient record from the selected list was excluded from the study, the next eligible record fulfilling the inclusion criteria was selected to replace the excluded record.

### 2.4. Data quality control

Data entries from 10% of the selected patient records were re-abstracted by the team facilitator immediately after the needed records were abstracted by the data extraction team using a systematic random sampling method. Once the files had been re-abstracted, any discrepancies between the data initially abstracted and the data that were re-abstracted were discussed, and the option that was unanimously agreed to be correct was selected. We have used guidelines for strengthening the reporting of observational studies in epidemiology (see Supplementary File 1, STROBE checklist).

### 2.5. Outcomes

The primary outcome was retention in HIV care, defined as regular attendance at appointments or engagement with the ART clinic at 3, 6, 12, 24, and ≥ 24 months after ART initiation. Secondary outcomes were LTFU, transferred out, stopped treatment, and death. LTFU was defined as a PWH whose medical records showed no evidence of any form of contact with a health facility for 30 or more days after the scheduled visit day. Transferred out is when a PWH moves to another ART clinic to continue receiving ART services. Stopped treatment was when a PWH stopped HIV treatment. We defined HIV viral suppression as having a viral load of less than 1,000 copies/mL after being on ART for ≥6 months as per national guidelines (37).

### 2.6. Sample size estimation

PS software was used to estimate the sample size (38). We hypothesized that the proportion of retention would be slightly higher after a test-and-treat period. An uncorrected chi-squared test was used to test the difference in proportions of retention between the two groups (before and after test-and-treat implementation), and the sample size was estimated accordingly with reference to a study conducted in Malawi (19). The proportion of retention BTT was 76.2%, and we expected a 3.7% increase in ATT (79.9%). We estimated requiring 6,688 records to achieve at least a 90% chance of detecting a difference (one-sided α = 0.05, allowing 5% type I error, conservative design effect of 2, power = 0.9).

### 2.7. Facility and participant sampling methods

We selected 45 health facilities [21 rural health centers (RHC), 16 urban health centers (UHC), and eight hospitals] across the 12 districts from 274 ART sites. In the initial step, the 274 ART sites were stratified into rural and urban sites, and then, a probability based on the number of PWH on ART at each health facility was used to select the required number of sites from each stratum.

### 2.8. Data analysis

The data were entered in REDCap and thereafter exported to STATA version 15 and R for standard data analysis. Data were described using frequency and percentages for categorical variables and medians (interquartile ranges) for continuous variables. To test for normality, the Shapiro–Wilk test was used. The Wilcoxon rank-sum test was used to ascertain the statistical difference between the two medians. A relationship between two categorical variables was determined using a chi-squared test. Multivariable logistic regression was used to examine the factors associated with retention using generalized estimating equations (GEE) via the logit link function to account for correlation structure within observations. The variables [cohort (ATT), age, sex, facility location, marital status, duration to ART initiation after HIV diagnosis, WHO stage, ART regimen, and BMI] in the adjusted analysis were selected based on previous literature (19, 26, 31, 32) and knowledge from HIV treatment and management experts. To ascertain statistical significance, a *p* value of <0.05 was used.

### 2.9. Ethics

Ethical approval was obtained from the Macha Research Trust Institution Review Board (IRB) and the Zambia National Health Research Authority (ZNHRA). This project was also reviewed in accordance with the Centers for Disease Control and Prevention’s (CDC) human research protection guidelines. In this study, we analyzed de-identified data from healthcare facilities.

## 3. Results

### 3.1. Characteristics of the study participants

**Figure 1:**
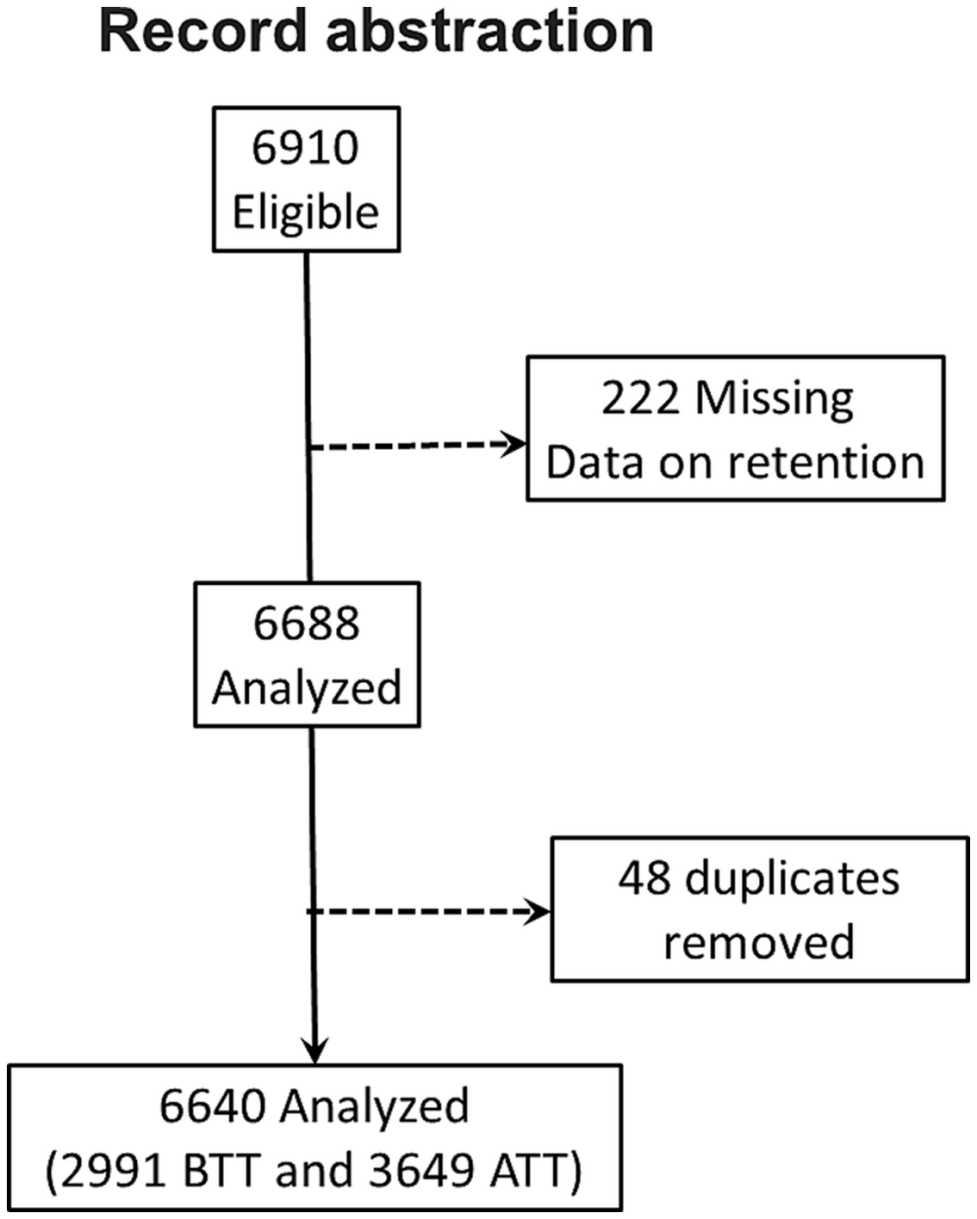
Consort diagram of record abstraction flow.

Owing to the anticipated potential presence of duplicates and missing information, we abstracted 6,910 records. After removing duplicates and records with missing information on the outcome variable, only 6,640 records were included in the analysis (Figure 1). We identified 2,991 (45.1%) records for PWH initiated on ART before test-and-treat (BTT) and 3,649 (54.9%) after test-and-treat (ATT) policies (Figure 1). The median age was 38 years [interquartile range (IQR), 31.0, 46.0], and participants in the BTT policy were significantly older, 40 vs. 37 years, *p* < 0.001. Women comprised 60.2% (*n* = 3,995) of PWH in the study sample. The majority of the participants, BTT (70.5%, *n* = 2,110) and ATT (55.9%, *n* = 2040), were from urban areas. There were more married individuals than those who were never married, divorced, or widowed in both the BTT (67.8%, *n* = 1813) and ATT (62.9%, *n* = 2022) cohorts. The baseline body mass index (BMI) was significantly higher among participants in the ATT than the BTT (21.6 vs. 21.3 kg/m^2^, *p* < 0.001). BTT participants took 14 days (IQR: 0, 32) to be initiated on ART from the time of HIV diagnosis compared with the ATT cohort that took 0 days (IQR: 0, 1), *p* = 0.001. A high proportion of the participants were taking non-nucleotide reverse transcriptase inhibitors (NNRTIs) at baseline (99.7% BTT and 77.6% ATT) and currently (71.4% BTT and 56.8% ATT). At baseline, the majority of patients had WHO stage I HIV, with a higher proportion of patients in the ATT cohort with Stage I (83.9%) vs. those in the BTT cohort (77.2%; Table 1).

**Table 1:** Demographic and clinical characteristics sorted by treatment policy status.

	Before test-and-treatment		After test-and-treatment			Overall
Characteristic	*N* = 2,991		*N* = 3,649		*p* value	
Current age^**^	2,991	34, 40, 47	3,649	30, 37, 45	<0.001^w^	31, 38, 46
Sex^*^	2,991		3,649		0.531^c^	
Men		1,179 (39.4)		1,466 (40.2)		2,645 (39.8)
Women		1,812 (60.6)		2,183 (59.8)		3,995 (60.2)
Facility location^*^	2,991		3,649		<0.001^c^	
Urban		2,110 (70.5)		2,040 (55.9)		4,150 (62.5)
Rural		881 (29.5)		1,609 (44.1)		2,490 (37.5)
Marital status^*^	2,673		3,213		<0.001^c^	
Married		1,813 (67.8)		2,022 (62.9)		3,835 (65.2)
Never married		331 (12.4)		541 (16.9)		872 (14.8)
Divorced		297 (11.1)		434 (13.5)		731 (12.4)
Widowed		232 (8.7)		216 (6.7)		448 (7.6)
Baseline BMI^**^	2,732	19.2, 21.3, 24.0	3,086	19.5, 21.6, 24.2	<0.001^w^	19.4, 21.5, 24.2
BMI at 6–12 months^**^	2,547	19.6, 21.6, 24.4	2,627	19.7, 21.8, 24.5	0.161^w^	19.7, 21.7, 24.4
BMI at ≥13 months^**^	2,669	19.8, 22.0, 25.0	2,543	19.9, 22.1, 25.1	0.541^w^	19.8, 22.0, 25.0
Duration to ART initiation^**^, days^x^	2,988	0, 14, 32	3,649	0, 0, 1	<0.001^w^	0, 0, 16
Baseline ART-based regimen^*^	2,969		3,649		<0.001^c^	
NNRTIs (NVP & EFV)		2,959 (99.7)		2,833 (77.6)		5,792 (87.5)
INSTI (DTG)		0 (0.0)		781 (21.4)		781 (11.8)
PI (LPV/r&ATV/r)		10 (0.3)		35 (1.0%)		45 (0.7)
Current ART-based regimen^*^	2,990		3,649		<0.001^c^	
NNRTIs (NVP& EFV)		2,135 (71.4)		2,071 (56.8)		4,206 (63.3)
INSTI (DTG)		806 (27.0)		1,537 (42.1)		2,343 (35.3)
PI (LPV/r & ATV/r)		49 (1.6)		41 (1.1)		90 (1.4)
Baseline WHO staging^*^	2,660		3,160		<0.001^c^	
Stage 1		2054 (77.2)		2,652 (83.9)		4,706 (80.8)
Stage 2		331 (12.4)		325 (10.3)		656 (11.3)
Stage 3		240 (9.0)		160 (5.1)		400 (6.9)
Stage 4		35 (1.3)		23 (0.7)		58 (1.0)
Current WHO staging^*^	2,890		3,440		0.238^f^	
Stage 1		2,841 (98.3)		3,402 (98.9)		6,243 (98.6)
Stage 2		28 (1.0)		23 (0.6)		51 (0.8)
Stage 3		18 (0.6)		13 (0.4)		31 (0.5)
Stage 4		3 (0.1)		2 (0.1)		5 (0.1)

Data are presented as ^*^n (%) or ^**^Q1MQ3, ^x^Period between enrolment and ART initiation, *n* number of no-missing values; ART, Antiretroviral therapy; Q1MQ3 25th 50th 75th percentiles; *n* (%), frequency and percentage; BMI, Body mass index; kg/m^2^, kilogram per meter squared; NNRTI, Non-nucleoside/nucleotide reverse transcriptase inhibitor (EFV = efavirenz and NVP=Nevirapine); PI, Protease inhibitor (LPV/*r* = lopinavir/ritonavir and ATV/*r* = atazanavir/ritonavir); INSTI, Integrase strand transfer inhibitor (DTG = dolutegravir); WHO, World Health Organization; ^w^Wilcoxon rank sum test; ^c^chi-square test; ^f^Fisher’s exact test.

### 3.2. Immunovirological outcomes by cohort

The overall prevalence of CD4 ≥ 500 cells/μL was 22.9, 37.9, and 47.8% at baseline, 6 months, and 12 months, respectively. Similarly, an increase in the proportion of patients with viral suppression (VL <1,000 copies/μL) was observed at different time points: 6 months (80.5%), 12 months (92.4%), and 24 months (94.6%).

At baseline, a significantly higher proportion of participants in the ATT cohort had CD4 ≥ 500 cells/μL than BTT (26.6 vs. 19.5%, *p* < 0.001). However, at 12 months, a higher proportion of participants in BTT than ATT had CD4 ≥ 500 cells/μL, 49.6 vs. 45.1%, *p* = 0.018. A higher proportion of viral suppression was observed among the participants in the BTT cohort at 6 months (86.1 vs. 77.4%, *p* < 0.001) and 12 months (93.6 vs. 91.5%, *p* = 0.030). At 24 months, the viral load categories were comparable (Table 2).

**Table 2:** Immunovirological outcomes by cohort.

Characteristic	Before test-and-treat		After test-and-treat		*p* value	Overall
	*n*		*n*			
Baseline CD4 absolute count (cells/μL)^*^	2,340		2,133		<0.001	
< 350		1,434 (61.3)		1,147 (53.8)		2,581 (57.7)
350–499		450 (19.2)		418 (19.6)		868 (19.4)
≥ 500		456 (19.5)		568 (26.6)		1,024 (22.9)
CD4 absolute count (cells/μL)^*^ at 6 months	1,962		1,594		0.061	
< 350		795 (40.5)		624 (39.1)		1,419 (39.9)
350–499		456 (23.2)		334 (21.0)		790 (22.2)
≥ 500		711 (36.2)		636 (39.9)		1,347 (37.9)
CD4 absolute count (cells/μL)^*^ at 12 months	2,289		1,527		0.018	
<350		648 (28.3)		484 (31.7)		1,132 (29.7)
350–499		506 (22.1)		355 (23.2)		861 (22.5)
≥500		1,135 (49.6)		688 (45.1)		1,823 (47.8)
Viral load (copies/μL)^*^ at 6 months	591		1,048		<0.001	
<1,000		509 (86.1)		811 (77.4)		1,320 (80.5)
≥1,000		82 (13.9)		237 (22.6)		319 (19.5)
Viral load (copies/μL)^*^ at 12 months	1,238		1,736		0.030	
<1,000		1,159 (93.6)		1,588 (91.5)		2,747 (92.4)
≥1,000		79 (6.4)		148 (8.5)		227 (7.6)
Viral load (copies/μL)^*^ at 24 months	2,430		2,385		0.210	
<1,000		2,290 (94.2)		2,267 (95.1)		4,557 (94.6)
≥1,000		140 (5.8)		118 (4.9)		258 (5.4)

Data are presented as ^*^n (%). *n*, Number of no-missing values; *n* (%), Frequency and percentage.

### 3.3. Overall retention in HIV care

At the time of data abstraction, 5,543 (83.4%) were actively on ART, 13 (0.2%) had stopped ART, 412 (6.2%) were transferred out, 621 (9.4%) were LTFU, and 51 (0.8%) were dead (Table 3). There was a statistically higher proportion of retention on ART for the ATT cohort as compared to the BTT cohort (90.5 vs. 74.8%, *p* < 0.001), while attrition was generally high in the BTT cohort as compared to the ATT cohort: death (1.4 vs. 0.2%, *p* < 0.001), stopped treatment (0.3 vs. 0.1%, *p* < 0.001), transferred out (9.9 vs. 3.2%, *p* < 0.001), and LTFU (13.5 vs. 5.9%, *p* < 0.001).

**Table 3:** Retention and attrition characteristics sorted by cohort type.

	*n* (%)	Cohort, *n* (%)		*p* value
		BTT	ATT	
Clinical outcome				<0.001
*Retained in care and on treatment*	5,543 (83.5)	2,238 (74.8)	3,305 (90.6)	
*Stopped treatment*	13 (0.2)	9 (0.3)	4 (0.1)	
*Transferred out*	412 (6.2)	296 (9.9)	116 (3.2)	
*Lost to follow-up*	621 (9.4)	405 (13.5)	216 (5.9)	
*Died*	51 (0.8)	43 (1.4)	8 (0.2)	

*n*, Number of no-missing values; *n* (%), Frequency and percentage; BTT, Before test-and-treat; ATT, After test-and-treat.

### 3.4. Retention at different time points by cohort

**Figure 2:**
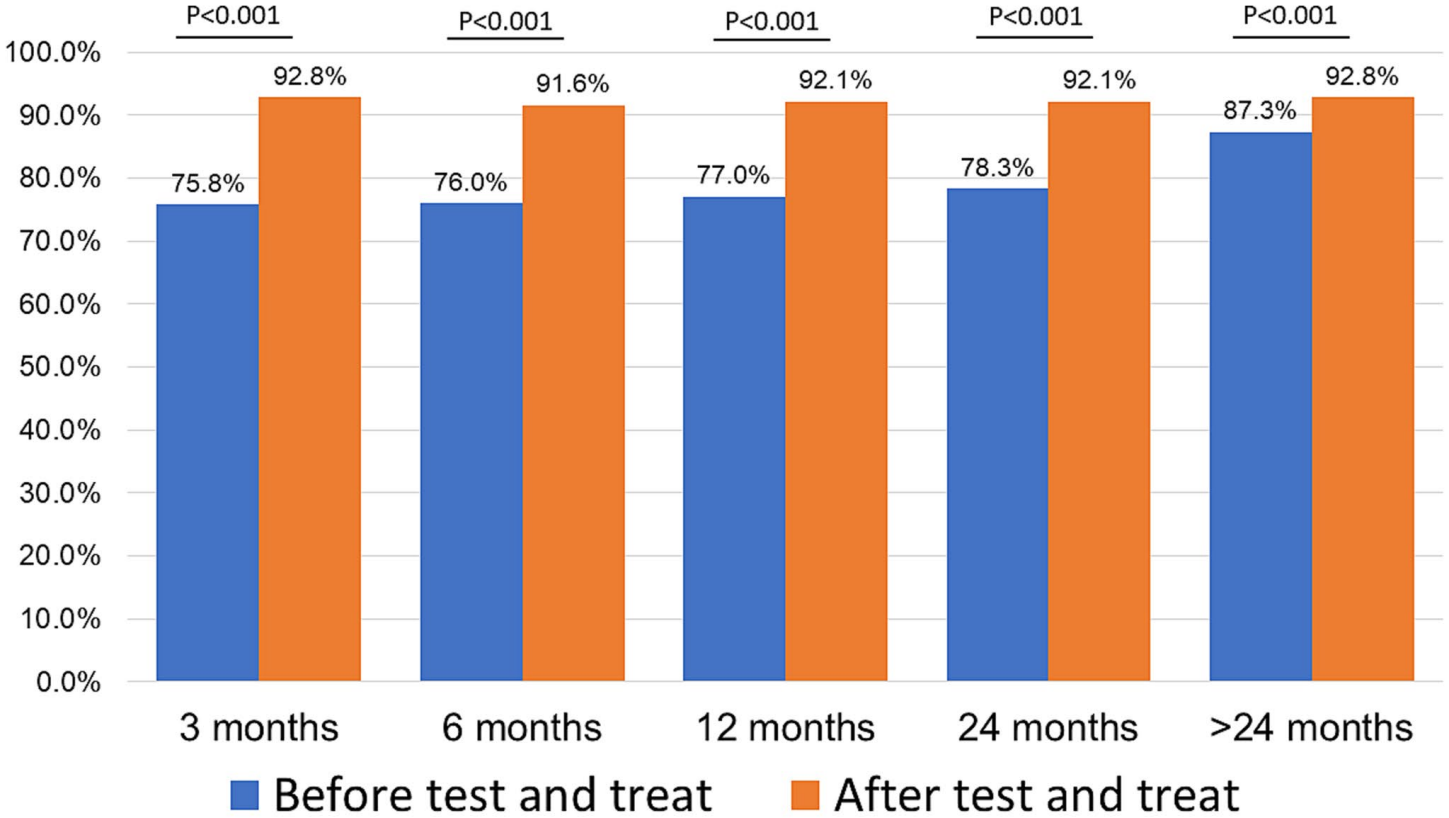
Retention rate at different time periods by cohort.

Retention was higher in the ATT cohort as compared to the BTT cohort (Figure 2) at 3 months (92.8 vs. 75.8%, *p* < 0.001), 6 months (91.6 vs. 76.0%, *p* < 0.001,), 12 months (92.1 vs. 77.0%, *p* < 0.001), 24 months (92.1 vs. 78.3%, *p* < 0.001), and > 24 months (92.8 vs. 87.3%, *p* < 0.001), that is, all time points.

### 3.5. Retention in HIV care at ≥24 months sorted by demographic and clinical characteristics

#### 3.5.1. Combined analysis (BTT and ATT)

Over half of the patients (59.6%, *n* = 3,305) who were retained in care were from the ATT cohort, whereas over two-thirds of the patients (40.4%, *n* = 2,238) were not retained in care were from the BTT cohort. The individuals who were retained in HIV care were significantly older than the ones who were not retained (35.1 vs. 33.1 years, *p* < 0.001). There was a higher proportion of urban participants who were not retained (70.1%) compared to those retained in HIV care. Conversely, there was a higher proportion of rural participants who were retained in HIV care (39.0%) vs. not retained in care (29.9%). Baseline BMI (21.5 vs. 21.0 kg/m^2^) and CD4 count (306 vs. 283 cells/μL) were significantly higher among the participants who were retained in HIV care in comparison to non-retained ones, with a *p* value of <0.05. The duration from HIV diagnosis to initiation of treatment was significantly lower among the retained participants as compared with those who were not retained (0 vs. 6 days, *p* < 0.001). Equally, the viral load at 24 months among the retained individuals in HIV care was lower (0 vs. 20 copies/μL, *p* < 0.001). A higher proportion of the participants whose dolutegravir (DTG) was their baseline ART regimen were retained in HIV care compared to those who were not retained (12.7 vs. 7.1%, *p* < 0.001). Among those in the NNRTI group, the preponderance of them were not retained in care (92.3 vs. 86.6%). A significantly higher percentage of the baseline participants with WHO stage 1 were retained in HIV care compared to those who were not retained (81.4 vs. 78.0%, *p* < 0.001; Table 4).

**Table 4:** Demographic and clinical factors sorted according to retention status.

Characteristic		Retained in HIV care		*p* value
	*n* = 6,640	Yes, 5,543 (83.5)	No, 1,097 (16.5)	
Cohort	6,640			<0.001^c^
BTT		2,238 (40.4)	753 (68.6)	
ATT		3,305 (59.6)	344 (31.4)	
Age at enrolment in years, median (IQR)	6,640	35.1 (28.3, 42.4)	33.1 (26.6, 40.4)	<0.001^w^
Sex^*^	6,640			0.737^c^
Male		2,213 (39.9)	432 (39.4)	
Female		3,330 (60.1)	665 (60.6)	
Facility location^*^	6,640			<0.001^c^
Urban		3,381 (61.0)	769 (70.1)	
Rural		2,162 (39.0)	328 (29.9)	
Marital status^*^	5,886			0.122^c^
Married		3,225 (65.6)	610 (62.8)	
Never married		705 (14.3)	167 (17.2)	
Divorced		607 (12.4)	124 (12.8)	
Widowed		378 (7.7)	70 (7.2)	
Baseline BMI in kg/m^2^, median (IQR)	5,818	21.5 (19.4, 24.2)	21.0 (19.0, 23.5)	<0.001^w^
Baseline CD4 absolute count in cells/μL, median (IQR)	4,473	306 (172, 491)	283 (156, 439)	0.001^w^
Viral load at 24 months in copies/μL, median (IQR)	4,815	0 (0, 25)	20 (0, 94)	<0.001^w^
Duration to ART initiation in days ^x,^ median (IQR)	6,637	0 (0, 15)	6 (0, 21)	<0.001^w^
Baseline ART based regimen^*^	6,618			<0.001^c^
NNRTIs (NVP & EFV)		4,782 (86.6)	1,010 (92.3)	
INSTI (DTG)		703 (12.7)	78 (7.1)	
PI (LPV/r & ATV/r)		39 (0.7)	6 (0.6)	
Baseline WHO staging^*^	5,820			<0.001^c^
Stage 1		3,979 (81.4)	727 (78.0)	
Stage 2		552 (11.3)	104 (11.2)	
Stage 3		307 (6.3)	93 (10.0)	
Stage 4		50 (1.0)	8 (0.8)	

Data are presented as ^*^n (%) or median (IQR); ^x^ Period between enrolment and ART initiation; n, Number of no-missing values; ART, Antiretroviral therapy; IQR, Interquartile range; *n* (%), Frequency and percentage; BMI, Body mass index; kg/m^2^, kilogram per meter squared; NNRTI, Non-nucleoside/nucleotide reverse transcriptase inhibitor (EFV = efavirenz and NVP = Nevirapine); PI, Protease inhibitor (LPV/r = lopinavir/ritonavir and ATV/r = atazanavir/ritonavir); INSTI, Integrase strand transfer inhibitor (DTG = dolutegravir); WHO, World Health Organization; ^w^Wilcoxon rank sum test; ^c^chi-squared test.

### 3.6. Retention in HIV care at ≥24 months sorted by BTT and ATT

In the cohort BTT, the factors significantly associated with retention in HIV care were older age, facility location, marital status, higher baseline BMI and CD4 count, and being in baseline WHO stage 1. While ATT participants, older age, lower viral load at 24 months, and being initiated on ART on the same day of HIV diagnosis were the factors significantly associated with retention in HIV care (Supplementary Table 1).

### 3.7. Predictors of overall retention in HIV care

In the univariate analysis, there was a statistically significant association between retention and the following determinants: Participants in the ATT cohort were 3.72 times more likely to be retained in HIV care compared to individuals in BTT (OR: 3.72, 95%CI 3.44, 4.02). A year increase in age was significantly associated with 1% increased odds of being retained in HIV care [odds ratio (OR) 1.01; 95%CI 1.01, 1.02]. Participants from rural areas had a significantly higher probability (54%) of being retained in HIV care compared to those from urban areas (OR 1.54; 95%CI 1.42, 1.67). Individuals who had never been married had 22% reduced odds of being retained in HIV care compared to those who were married (OR 0.78; 95%CI 0.70, 0.85). An increase in the number of days from HIV diagnosis to ART initiation was significantly associated with a 1% reduced chance of being retained in HIV care. Participants who were in WHO stage 3 at baseline had 35% reduced odds of being retained in HIV care compared to the ones in stage 1 (OR 0.65; 95%CI 0.58, 0.73). Clients on a DTG and protease inhibitor (PI)-based regimen were 2.27 and 2.29 times more likely to be retained in HIV care relative to those on NNRTIs, respectively. A unit increase in baseline BMI was significantly associated with 3% increased odds of being retained in HIV care (OR 1.03; 95%CI 1.02, 1.04).

In the adjusted analysis, individuals from the ATT cohort were 3.67 times more likely to be retained in care. A unit increase in age was associated with a 1% increase in the odds of being retained in HIV care. Participants from rural areas had 32% higher odds of being retained in care than those from urban areas. Never married and divorced participants had 24 and 16% reduced odds of being retained in HIV care, respectively, compared to married ones. Baseline participants in WHO stage 2 had 18% increased odds of being retained in HIV care (OR 1.18; 95%CI 1.05, 1.32). However, baseline participants with WHO stage 3 had 23% reduced odds of being retained in HIV care. A unit increase in BMI increased the chance of being retained in care by 2% (Table 5).

**Table 5:** Crude and adjusted analyses of factors associated with retention.

Characteristic	Crude analysis				Adjusted analysis			
		95%CI				95%CI		
	OR	Lower	Upper	*p* value	OR	Lower	Upper	*p* value
Cohort								
BTT	Ref							
ATT	3.72	3.44	4.02	<0.001	3.67	3.36	4.01	<0.001
Age at ART enrolment in years	1.01	1.01	1.02	<0.001	1.01	1.01	1.02	<0.001
Sex								
Male	Ref							
Female	0.96	0.90	1.04	0.323	1.01	0.93	1.10	0.801
Facility location								
Urban	Ref							
Rural	1.54	1.42	1.67	<0.001	1.32	1.22	1.44	<0.001
Marital status								
Married	Ref							
Never married	0.78	0.70	0.85	<0.001	0.76	0.68	0.85	<0.001
Divorced	0.91	0.82	1.02	0.094	0.84	0.75	0.94	<0.001
Windowed	1.07	0.93	1.23	0.319	1.05	0.90	1.23	0.504
Duration to ART initiation in days^x^	0.99	0.99	0.99	<0.001	1.00	1.00	1.00	0.500
Baseline WHO staging								
Stage 1	Ref							
Stage 2	1.07	0.96	1.20	0.234	1.18	1.05	1.32	<0.001
Stage 3	0.65	0.58	0.73	<0.001	0.77	0.68	0.88	<0.001
Stage 4	1.13	0.79	1.62	0.497	1.41	0.96	2.07	0.079
Baseline ART-based regimen								
NNRTIs (NVP & EFV)	Ref							
INSTI (DTG)	2.27	1.96	2.63	<0.001	0.97	0.83	1.14	0.742
PI (LPV/r&ATV/r)	2.29	1.20	4.37	0.012	1.64	0.79	3.40	0.186
Baseline BMI in kg/m^2^	1.03	1.02	1.04	<0.001	1.02	1.01	1.03	<0.001

CI, Confidence interval; OR, Odds ratio; ^x^ Period between enrolment and ART initiation; ART, Antiretroviral therapy; BMI, Body mass index; kg/m^2^, Kilogram per meter squared; NNRTI, Non-nucleoside/nucleotide reverse transcriptase inhibitor (EFV = efavirenz and NVP = Nevirapine); PI, Protease inhibitor (LPV/r = lopinavir/ritonavir and ATV/r = atazanavir/ritonavir); INSTI, Integrase strand transfer inhibitor (DTG = dolutegravir).

### 3.8. Predictors of retention in HIV care among individuals (BTT and ATT)

In the BTT participant group, factors significantly associated with retention upon adjusted analysis included receiving care at a rural health facility (OR 1.54; 95%CI 1.21, 1.94) and being never married (OR 0.70; 95%CI 0.52, 0.95). In contrast, for the ATT cohort, factors linked to ART retention were increasing age (OR 1.02; 95%CI 1.01, 1.04) and being in WHO stage 2 (OR 2.32; 95%CI 1.20, 4.52). See Supplementary Table 2.

## 4. Discussion

This study aimed to determine the differences in retention rates and clinical outcomes for adult PWH enrolled on ART, BTT, and ATT policies. Participants who initiated ART and were enrolled in care after the test-and-treat policy had better retention in HIV care and health outcomes than those enrolled before the test-and-treat program. Our finding showed a higher prevalence of retention under ATT than what was previously reported in SSA (14) and Haiti (36); the variations could result from the fact that our study was conducted recently, and many other interventions have been aimed at improving retention in HIV care. Moreover, the majority of the participants in our project were on an INSTI-based regimen, which has shown higher tolerability than the other ART regimens (39). Retention was significantly associated with cohort (ATT), increasing baseline age, facility location (rural participants), marital status (never married and divorced), increasing baseline body mass index (BMI), and baseline WHO staging (2 and 3).

The evaluation results suggest that the universal and rapid test-and-treat implementation policy effectively retained clients in care. However, the clinical and immunological outcomes were better than BTT based on the studies conducted in Nigeria (40) and Zambia (20). Moreover, our follow-up period was longer than that in studies conducted in Nigeria and Lusaka, Zambia (2 years vs. 1 year). Retention was generally higher in the ATT cohort than in the BTT cohort, with an average retention rate of over 90%. However, the overall retention rate in the province was 83%. This is higher than reported in some African studies (41, 42) and recently in Lusaka, Zambia (20).

The enrollment age was younger for the ATT cohort than the BTT cohort, which is expected to indicate an effective and well-implemented policy. This was even evident with an average ART initiation turn-around time of 0 days compared to the BTT cohort, where the average ART initiation turn-around time was 14 days. This translated into good treatment outcomes, including clinical and immunological outcomes. For example, compared to the BTT cohort, a higher proportion of clients in the ATT cohort were in stage 1 of the WHO stage, had higher baseline CD4 counts, better attrition factors, and their BMI improved remarkably. However, the viral suppression and CD4 count seemed to be better before the test-and-treat policy. This could be because individuals under the ATT policy were relatively younger than those in BTT, and it has been well-studied that young people struggle to achieve viral suppression (43–45).

The majority of clients in the ATT cohort were retained on ART and still alive, and very few had stopped treatment, transferred out, lost to follow-up, or died. Our findings align with several clinical trials that assessed rapid universal treatment (46–49) and demonstrated that initiating individuals on ART immediately after an HIV diagnosis not only effectively retains PWH in care in real world settings but also enhances clinical outcomes, such as reducing mortality. On the contrary, US researchers observed similar rates of loss to follow-up between those who began ART on the same day and those who started based on their CD4 counts (13). These differences might be attributed to the varying sample sizes, with our study having greater statistical power. Additionally, recent interventions targeting a reduction in HIV loss to follow-up could also account for this discrepancy.

Mortality was significantly reduced during the ATT phase compared to the time before the universal test-and-treat policy, which is in line with what was observed in Ethiopia (50). One possible reason for this may be that, under BTT policies, most individuals had to reach an advanced WHO clinical stage before initiating ART (40, 51). Similarly, we observed that a greater number of participants were in the more severe WHO clinical stages before the implementation of the test-and-treat policy. The recipients of care ATT were younger than those BTT, which could partly explain the differences in death rates.

Our findings are not just the mere effect of implementing the test-and-treat policy among PWH; this program required implementing several programmatic adjustments, financial support, and increasing human resources to enhance care and support all structures involved in the treatment cascade of PWH, including counseling and testing services. In the real world, testing and treating all PWH to achieve viral suppression is possible when the whole ART cascade of care is enhanced.

### 4.1. Limitations of the study

Missing data led to delays in data abstraction. Some characteristics, such as viral load and CD4 count, were missing. We did not collect data on alcohol consumption, HIV disclosure status, year of enrollment for each participant, distance to the health facility, or adherence counseling, which could have helped explain retention rates among our study participants. Additionally, we could not get the patients’ perspectives regarding UTT policy. However, the study was able to quantify retention in HIV care and clinical outcomes before and after the universal test-and-treat policy.

### 4.2. Strengths of the study

We used a large sample size in our study compared to similar studies. Hence, this study was highly powered, and the results may be applicable to PWH living in Zambia. The methodology and statistical analysis were rigorous. The multicenter model and sampling methods used strengthened the study and minimized bias, providing a comprehensive overview of the whole program in the province.

## 5. Conclusion

Our data strongly indicate that the test-and-treat policy implemented in 2016 was significantly more effective in improving retention in HIV care and treatment compared to the BTT period. Additionally, clinical outcomes (alive and on treatment, transferred out of the facility, lost to follow-up, stopped treatment, and death) improved remarkably to acceptable levels in the ATT cohort. Factors that significantly influenced retention included the treatment policy, older age, location of the facility, not being married, high baseline BMI, and worse WHO stages. Hence, interventions targeting young individuals, urban care recipients, non-married people, and those with symptomatic WHO stages and a low BMI are encouraged.

## Data availability statement

The raw data supporting the conclusions of this article will be made available by the authors, without undue reservation.

## Ethics statement

Ethical approval was obtained from the Macha Research Trust Institution Review Board (IRB), and the Zambia National Health Research Authority (ZNHRA). This project was also reviewed in accordance with the Centers for Disease Control and Prevention (CDC) human research protection procedures. In this study, we analyzed de-identified data from healthcare facilities. The ethics committee/institutional review board waived the requirement of written informed consent for participation from the participants or the participants’ legal guardians/next of kin because the study was a retrospective study.

## Author contributions

BH and SMa drafted the manuscript. BH and IF conducted all statistical analyses. BH, SMu, LS, MS, JM, SKu, SKa, CC, IF, CB, and SMa participated in data collection. BM, KM, and NK guided study design and draft review. All authors contributed to the article and approved the submitted version.
