# Supplementary material for: HIV test-and-treat policy improves clinical outcomes in Zambian adults from Southern Province: a multicenter retrospective cohort study
Source: Front Public Health. 2023 Oct 11;11:1244125. doi: 10.3389/fpubh.2023.1244125 (PMC10600392; doi:10.3389/fpubh.2023.1244125)
Supplement: Supplementary file 1 [file Table_1.DOCX]

Supplementary Tables

| **Supplementary Table 1. Demographic and clinical factors sorted according to cohort and retention status** | | | | | | |  |  |
| --- | --- | --- | --- | --- | --- | --- | --- | --- |
|  | **BTT** | | | | **ATT** | | | |
| **Characteristic** | **N=2991** | **Retained in HIV Care** | | **p-value** | **N=3649** | **Retained in HIV Care** | | **p-value** |
|  |  | **Yes, 2238 (74.8%)** | **No, 753 (25.2 %)** |  |  | **Yes, 3305 (90.6%)** | **No, 344 (9.4%)** |  |
| **Age at enrolment** in years, median (IQR) | 2991 | 35.1 (28.8, 41.8) | 33.3 (26.7, 40.4) | **<0.001** | 3649 | 35.1 (28.0, 42.8) | 31.8 (26.4, 40.7) | **0.001** |
| **Sex*** | 2991 |  |  | 0.294 | 3649 |  |  | 0.079 |
| Male |  | 870 (38.9) | 309 (41.0) |  |  | 1343 (40.6) | 123 (35.8) |  |
| Female |  | 1368 (61.1) | 444 (59.0) |  |  | 1962 (59.4) | 221(64.2) |  |
| **Facility location*** | 2991 |  |  | **<0.001** |  |  |  | 0.881 |
| Urban |  | 1532 (68.5) | 578 (76.8) |  | 3649 | 1849 (56.0) | 191(55.5) |  |
| Rural |  | 706 (31.6) | 175 (23.2) |  |  | 1456 (44.1) | 153 (44.5) |  |
| **Marital status*** | 2991 |  |  | **0.008** |  |  |  | 0.323 |
| Married |  | 1387 (69.1) | 426 (64.0) |  |  | 1838 (63.2) | 184 (60.3) |  |
| Never married |  | 226 (11.3) | 105 (15.8) |  |  | 479 (16.5) | 62 (20.3) |  |
| Divorced |  | 215(10.7) | 82(12.3) |  |  | 392 (13.5) | 42 (13.8) |  |
| Widowed |  | 179(8.7) | 53 (8.0) |  |  | 199 (6.8) | 17 (5.6) |  |
| **Baseline BMI** in kg/m2, median (IQR) | 2732 | 21.4 (19.3, 24.2) | 20.9 (18.9, 23.5) | **0.002** | 3086 | 21.6 (19.5, 24.3) | 19.3 (17.9, 23.4 | 0.053 |
| **Baseline CD4 absolute count** in cells/µl, median (IQR) | 2340 | 289 (160, 458) | 271 (151, 426) | 0.056 | 2133 | 326 (179, 518) | 339 (177, 463) | 0.369 |
| **Viral load at 24 months** in copies/µl, median (IQR) | 2430 | 0 (0, 26) | 20 (0, 111) | **<0.001** | 1736 | 0 (0, 24) | 20 (0, 52) | **0.001** |
| **Duration to ART initiation** in days ^x,^ median (IQR) | 2988 | 14 (0, 34) | 14 (0, 28) | 0.812 | 3649 | 0 (0, 0) | 0 (0, 6) | **0.001** |
| **Baseline ART based regimen*** | 2969 |  |  | 0.701 | 3649 |  |  | 0.757 |
| NNRTIs (NVP & EFV) |  | 2211 (99.64) | 748 (99.3) |  | 3649 | 2,571 (77.8) | 262 (76.2) |  |
| INSTI (DTG) |  |  |  |  |  | 703 (21.3) | 78 (22.7) |  |
| PI (LPV/r & ATV/r) |  | 8 (0.36) | 2 (0.27) |  |  | 31 (0.94) | 4 (1.16) |  |
| **Baseline WHO staging*** | 2660 |  |  | **0.045** | 3160 |  |  | **0.015** |
| Stage 1 |  | 1557 (78.1) | 497 (74.6) |  |  | 2422 (83.9) | 230 (86.5) |  |
| Stage 2 |  | 241 (12.1) | 90 (13.5) |  |  | 311 (10.8) | 14 (5.3) |  |
| Stage 3 |  | 166 (8.32) | 74 (11.1) |  |  | 141 (4.9) | 19 (7.1) |  |
| Stage 4 |  | 30 (1.50) | 5 (0.75) |  |  | 20 (0.7) | 3 (1.1) |  |
| Note: data are presented as *n (%) or median (IQR), ^x^ Period between enrolment and ART initiation, n number of no-missing values, ART antiretroviral therapy, IQR interquartile range, n (%)- frequency and percentage, BMI- body mass index, kg/m2- kilogram per meter squared, NNRTI non-nucleoside/nucleotide reverse transcriptase inhibitor (EFV=efavirenz and NVP=Nevirapine), PI Protease inhibitor (LPV/r=lopinavir/ritonavir and ATV/r=atazanavir/ritonavir), INSTI integrase strand transfer inhibitor (DTG=dolutegravir), WHO world health organization, ^w^ Wilcoxon rank sum test, ^c^ chi-square test. | | | | | | | | |

| Supplementary Table 2. Crude and adjusted analyses of factors associated with retention in HIV care among participants BTT and ATT | | | | | | | | | | | | | | | | |
| --- | --- | --- | --- | --- | --- | --- | --- | --- | --- | --- | --- | --- | --- | --- | --- | --- |
| **Variable** | **BTT cohort** | | | | | | | | **ATT cohort** | | | | | | | |
|  | **Crude analysis** | | | | **Adjusted analysis** | | | | **Crude analysis** | | | | **Adjusted analysis** | | | |
|  |  | **95%CI** | |  |  | **95%CI** | |  |  | **95%CI** | |  |  | **95%CI** | |  |
|  | **OR** | **Lower** | **Upper** | **P value** | **OR** | **Lower** | **Upper** | **P value** | **OR** | **Lower** | **Upper** | **P value** | **OR** | **Lower** | **Upper** | **P value** |
| **Age at ART enrolment** in Years | 1.01 | 1.00 | 1.02 | **0.030** | 1.01 | 1.00 | 1.01 | 0.238 | 1.02 | 1.01 | 1.03 | **0.001** | 1.02 | 1.01 | 1.04 | **0.007** |
| Sex |  |  |  |  |  |  |  |  |  |  |  |  |  |  |  |  |
| Male | ref |  |  |  | ref |  |  |  | ref |  |  |  | ref |  |  |  |
| Female | 1.09 | 0.92 | 1.29 | 0.294 | 1.05 | 0.85 | 1.30 | 0.632 | 0.81 | 0.64 | 1.02 | 0.079 | 0.90 | 0.65 | 1.26 | 0.556 |
| **Facility location** |  |  |  |  |  |  |  |  |  |  |  |  |  |  |  |  |
| Urban | Ref |  |  |  | ref |  |  |  | ref |  |  |  |  |  |  |  |
| Rural | 1.52 | 1.26 | 1.84 | **<0.001** | 1.54 | 1.21 | 1.94 | **<0.001** | 0.98 | 0.79 | 1.23 | 0.881 | 1.03 | 0.76 | 1.41 | 0.843 |
| **Marital status** |  |  |  |  |  |  |  |  |  |  |  |  |  |  |  |  |
| Married | Ref |  |  |  | Ref |  |  |  | ref |  |  |  | ref |  |  |  |
| Never married | 0.66 | 0.51 | 0.85 | **0.002** | 0.70 | 0.52 | 0.95 | **0.021** | 0.77 | 0.57 | 1.05 | 0.099 | 0.92 | 0.62 | 1.38 | 0.703 |
| Divorced | 0.80 | 0.61 | 1.06 | 0.125 | 0.81 | 0.59 | 1.09 | 0.168 | 0.93 | 0.66 | 1.33 | 0.706 | 0.93 | 0.60 | 1.46 | 0.763 |
| Windowed | 1.04 | 0.75 | 1.44 | 0.825 | 1.14 | 0.77 | 1.70 | 0.505 | 1.17 | 0.70 | 1.97 | 0.548 | 0.85 | 0.45 | 1.58 | 0.601 |
| **Duration to ART initiation in days** ^x^ | 1.00 | 0.99 | 1.00 | 0.389 | 1.00 | 1.00 | 1.00 | 0.734 | 0.99 | 0.99 | 0.99 | **0.032** | 0.99 | 0.99 | 1.00 | 0.107 |
| **Baseline WHO staging** |  |  |  |  |  |  |  |  |  |  |  |  |  |  |  |  |
| Stage 1 | Ref |  |  |  | ref |  |  |  | ref |  |  |  | ref |  |  |  |
| Stage 2 | 0.85 | 0.66 | 1.11 | 0.241 | 0.97 | 0.72 | 1.30 | 0.834 | 2.10 | 1.21 | 3.66 | **0.008** | 2.32 | 1.20 | 4.52 | **0.012** |
| Stage 3 | 0.72 | 0.53 | 0.96 | **0.025** | 0.82 | 0.59 | 1.13 | 0.227 | 0.70 | 0.43 | 1.16 | 0.168 | 0.61 | 0.35 | 1.05 | 0.073 |
| Stage 4 | 1.92 | 0.74 | 4.96 | 0.181 | 2.15 | 0.74 | 6.21 | 0.159 | 0.63 | 0.19 | 2.15 | 0.463 | 0.58 | 0.17 | 2.00 | 0.385 |
| **Baseline ART based regimen** |  |  |  |  |  |  |  |  |  |  |  |  |  |  |  |  |
| NNRTIs (NVP & EFV) | Ref |  |  |  | Ref |  |  |  | ref |  |  |  | ref |  |  |  |
| INSTI (DTG) | 1 |  |  |  | 1 |  |  |  | 0.92 | 0.70 | 1.20 | 0.531 | 0.93 | 0.64 | 1.34 | 0.699 |
| PI (LPV/r&ATV/r) | 1.35 | 0.29 | 6.39 | 0.702 | 1 |  |  |  | 0.79 | 0.28 | 2.25 | 0.659 | 0.67 | 0.154 | 3.00 | 0.609 |
| **Baseline BMI** in kg/m2 | 1.03 | 1.00 | 1.05 | **0.015** | 1.02 | 0.99 | 1.04 | 0.173 | 1.03 | 1.00 | 1.06 | 0.082 | 1.04 | 0.99 | 1.08 | 0.083 |
| Note: CI confidence interval, OR odds ratio, ^x^ Period between enrolment and ART initiation, ART antiretroviral therapy, BMI- body mass index, kg/m2- kilogram per meter squared, NNRTI non-nucleoside/nucleotide reverse transcriptase inhibitor (EFV=efavirenz and NVP=Nevirapine), PI Protease inhibitor (LPV/r=lopinavir/ritonavir and ATV/r=atazanavir/ritonavir), INSTI integrase strand transfer inhibitor (DTG=dolutegravir). | | | | | | | | | | | | | | | | |
